# The Skin Microbiome Profile of Contact Sports Athletes—Focus on Sexual Dimorphism and Athlete–Non-Athlete Differences

**DOI:** 10.3390/sports13090288

**Published:** 2025-08-26

**Authors:** Irina Kalabiska, Dorina Annar, Gergely Babszky, Matyas Jokai, Zoltan Borbas, Gergely Hajdu, Fanny Zselyke Ratz-Sulyok, Csilla Jang-Kapuy, Gergely Palinkas, Harjit Pal Bhattoa, Annamaria Zsakai

**Affiliations:** 1 Research Center for Sport Physiology, Hungarian University of Sports Science, Alkotas u. 44, 1123 Budapest, Hungaryhajdu.gergely@tf.hu (G.H.);; 2 Kozma Istvan Hungarian Wrestling Academy, Hollandi u. 8, 1213 Budapest, Hungary; 3 Physical Training, Regulation, Metabolism Programme, School of Doctoral Studies, Hungarian University of Sports Science, Alkotas u. 44, 1123 Budapest, Hungary; 4 Neuroscience and Human Biology Programme, Doctoral School of Biology, Eotvos Lorand University (ELTE), Pazmany P. s. 1/c, 1117 Budapest, Hungary; 5 Department of Biological Anthropology, Eotvos Lorand University (ELTE), Pazmany P. s. 1/c, 1117 Budapest, Hungary; annamaria.zsakai@ttk.elte.hu; 6 Department of Laboratory Medicine, Faculty of Medicine, University of Debrecen, Nagyerdei Blvd. 98, 4032 Debrecen, Hungary; harjit@med.unideb.hu; 7 Health Promotion and Education Research Team, Hungarian Academy of Sciences, Pazmany P. s. 1/c, 1117 Budapest, Hungary

**Keywords:** skin, microbiome, athletes, contact sports, sex differences, skin infection prevention

## Abstract

Background: Athletes’ skin is exposed to increased microbial challenges due to rigorous physical activity, perspiration, constant “skin-to-skin” contact, frequent showering, use of hygiene products, and environmental factors present in training settings. This study aims to characterize the skin microbiome communities of young wrestlers and kickboxers in comparison with their non-athlete age-peers. Methods: A total of 56 combat sport athletes (30 males and 26 females, mean age ± SD = 18.2 ± 1.5 years) and 25 non-athlete youths (control group: 13 males and 12 females, mean age ± SD = 19.8 ± 1.2 years) voluntarily consented to participate in the study conducted by our research team in 2023 and 2024. The skin microbiome analysis involved standardized sampling, DNA isolation, molecular sequencing, and bioinformatic analysis, thus enabling detailed characterization and comparison of the skin microbial community in contact sports athletes and the control group. Results: Our results revealed notable sexual dimorphism in the skin microbiome composition of youth. Males showed a higher relative abundance of bacterial genera associated with nosocomial infections and respiratory diseases, while females had more skin inflammation- and infection-related genera (relative abundances in males vs. in females: *Corynebacterium*—12.0 vs. 7.2; *Luteimonas*—4.4. vs. 1.4; *Paracoccus*—8.8 vs. 5.0; *Psychrobacter*—6.3 vs. 4.4; *Cutibacterium*—6.4 vs. 11.4; *Kocuria*—1.6 vs. 3.9; *Micrococcus*—5.8 vs. 8.5; *Pseudomonas*—1.2 vs. 3.4; *Streptococcus* 3.3 vs. 6.2). We also found skin microbiome differences between athletes and non-athletes in both sexes: wrestlers, who experience frequent skin-to-skin contact and wear less covering sportswear, had microbiome profiles distinct from both kickboxers and non-athletes (relative abundances in athletes vs. in non-athletes: *Psychrobacter*—7.3 vs. 0.4; *Staphylococcus* 9.5 vs. 18.5; predominance of genera by sports type: relative abundance of *Cutibacterium* and *Streptococcus* was higher in kickboxers, and relative abundance of *Acinetobacter*, *Enhydrobacter*, *Micrococcus*, and *Enhydrobacter* was higher in wrestlers). Bacteria linked to skin infections (e.g., *Aliterella*, *Arthrobacter*, and *Empedobacter*) were present in around 30% of wrestlers and kickboxers but were absent in the control group. Conclusions: These results underscore the heightened risk of skin infections in contact sports and highlight the importance of regular microbiome monitoring and hygiene protocols among young athletes.

## 1. Introduction

The preservation of athletes’ health and the investigation of the long-term effects of sports on the human body have received increasing global attention in recent years [1]. Although regular physical activity offers numerous physiological benefits, it also places considerable stress on the body, potentially affecting the immune and endocrine systems as well as the functionality of the microbiomes of the different body parts [2,3,4]. The skin, as the body’s primary barrier against external factors, is particularly vulnerable to the environmental effects associated with intense physical activity—e.g., tight or synthetic fabrics can cause pressure-induced irritation or contact with gym equipment (mats, outdoor terrain) can introduce bacteria or cause abrasions [5,6].

The skin microbiome is a complex community of microorganisms that is essential for maintaining skin homeostasis, modulating immune responses, and protecting against pathogens and environmental factors [7,8]. The microbial community’s composition varies across anatomical regions of the skin and is shaped by physiological factors such as moisture, sebum secretion, and pH levels. The predominant bacterial phyla include *Actinobacteria*, *Firmicutes*, *Proteobacteria*, and *Bacteroidetes*. The skin microbiome undergoes dynamic changes in response to factors such as age, sex, hygiene practices, and environmental conditions. These shifts are pronounced during adolescence, when hormonal fluctuations significantly influence microbial balance [9,10]—during puberty, hormonal shifts increase sebum production, which alters the skin’s microbial profile: the predominance of lipophilic microbes (e.g., *Cutibacterium* or *Corynebacterium*) increases.

Athletes’ skin is exposed to increased microbial challenges due to rigorous physical activity, perspiration, frequent showering and skin-to-skin contact, the use of hygiene products, and environmental factors present in training settings (e.g., locker rooms, mats, and equipment) [11]. These factors may alter the composition of the skin microbiome, affecting both its protective function against infections and its role in regulating inflammatory processes. Disruption of microbial balance (dysbiosis) may, over time, contribute to the development of chronic dermatological conditions and influence the overall well-being [12]. Consequently, research on skin microbiome is gaining growing importance in sports medicine and preventive health studies.

In contact sports, frequent skin-to-skin or skin-to-surface contact (whether direct or via shared environments) substantially increases the risk of microorganism transmission. Consequently, the incidence of infection-related dermatological conditions is higher among athletes in these disciplines than in non-athletes [13]. Wrestlers and judokas are prone to bacterial (e.g., *Staphylococcus aureus*, *S. epidermidis*, *S. saprophyticus*, or *Streptococcus pyogenes*), viral (e.g., *Herpes simplex virus*, *Human papillomavirus*, or *Molluscum contagiosum virus*), and fungal pathogens (e.g., *Trichophyton tonsurans*) skin infections. In particular, fungal infections can spread epidemically during training camps or competition periods and are often transmitted via asymptomatic carriers [13,14,15].

A focused analysis of wrestlers and kickboxers is warranted, given that their sports involve vigorous and recurrent physical contact, along with exposure to microtraumas, which can significantly affect the composition of skin microbial communities. The competition- and training-specific attributes of these disciplines—such as close physical contact, continuous exposures of skin surfaces, and frequent injuries—create a distinctive context for examining the dynamics of the skin microbiome in athletes of these contact sports. Furthermore, infection-associated dermatological conditions frequently reported in wrestling (e.g., tinea gladiatorum, impetigo, and herpes simplex) have a notable epidemiological relevance, making the assessment of these athletes’ microbial status important from both preventive and public health perspectives [16,17].

Wrestling can be characterized by a high-contact, high-friction environment for athletes that can disrupt the skin barrier and support the colonization of opportunistic microbes. Kickboxing, while it is still an intense type of sport, involves less direct skin contact and more protective gear, which may buffer the skin but also retain the moisture of the tissue. This type of sport also alters the skin microbial profile depending on textile type.

This study aims to characterize the skin microbiome communities of young wrestlers and kickboxers in comparison with their non-athlete age-peers, with special attention to sexual dimorphism, that is, examining potential differences in the composition and diversity of the skin microbiota between male and female participants. It was hypothesized that contact sports athletes would present a distinct skin microbiome profile compared to non-athletes, with sex-specific differences in bacterial diversity and abundance. The main reasons for the selection of the studied combat sports were the following: both wrestling and kickboxing are physically demanding, and their impact on the skin microbiome is supposed to differ significantly. Wrestling’s intense skin-to-skin contact and minimal clothing may create a fertile ground for microbial exchange and dysbiosis. In contrast, kickboxers’ protective gear and striking-based movements reduce the frequency of direct contact, but introduce textile-related microbiome challenges.

## 2. Materials and Methods

### 2.1. Subjects

Skin microbial profile of 56 elite athletes (combat sports athletes—30 males and 26 females, aged between 16 and 22 years, mean age ± SD = 18.2 ± 1.5 years) and 25 non-athlete youths (control group; 13 males and 12 females, aged between 18 and 22 years, mean age ± SD = 19.8 ± 1.2 years, with low level of physical activity: training any kind of sports less than 3 h/week, no participation in organized sports) were examined in a cross-sectional study in 2023. The athlete cohort consisted of elite kickboxers (16 males and 11 females) and elite wrestlers (14 males and 15 females)—training more than 8 h/week in the competition season. Microbial samples were collected only from those participants who followed the common hygienic practices (having showers/baths less than twice a day, using a personal towel, following the common gear policies during sports activities). Data on the level of physical activity, hygienic habits were collected during the personal interviews conducted by the anthropologists of the research team.

The minimum required sample size (for males vs. females and athletes vs. non-athletes comparisons) was calculated by using a statistical power of 80%, a significance level of 0.05, and an expected effect size of 0.8. The minimum required sample size per subgroup in the planned comparisons was 26 participants; therefore, a minimum of 52 participants were required for the analyses. A total of 90 volunteers were recruited, and finally 81 participants were studied (43 males and 38 females; 56 athletes and 25 non-athletes; and 9 invited participants could not be studied due to illnesses, infections, or other activities); all the subgroups were bigger than 26, with the exception of non-athletes—25 non-athletes were studied. The studied athletes were members of the Hungarian national teams.

Participants with recent antibiotic use (within the last three months), chronic skin conditions, or systemic diseases that could influence skin microbiome composition were excluded from the microbiome study. The participants were asked not to use topical antimicrobial agents and to maintain their usual hygiene routines for at least one week before the examinations. Skin microbiome sampling was performed during the morning hours, with athletes specifically requested not to shower on the morning of sample collection day to minimize external influences on the skin microbiome and to reduce confounding from hygiene routines.

### 2.2. Sampling

Skin microbiome analysis was conducted following a standardized protocol [4]. Samples were collected from the skin of wrestlers, kickboxers, and non-athletes. The specific body site (lower arm anterior region—due to practical, hygienic and anatomical considerations: flat surface, minimal body hair, less contact with clothing, reduced sebaceous activity, less exposure to disinfectants—stable and not specific microbial community of the skin, ease of replication and common site for microbial sampling, reduced moisture-related microbial overgrowth) was selected in accordance with the research protocol, ensuring consistent sampling across all the participants. Prior to sampling, the skin surface was not cleaned in order to preserve the natural composition of the microbial community. Samples were collected by sterile swabs and immediately placed in a cooled storage container to prevent DNA degradation. The samples were stored at −20 °C for no longer than 2 weeks before the microbiome analyses.

### 2.3. Microbial Analyses

DNA was isolated by the QIAmp DNA Blood Mini Kit (Qiagen, Beverly, MA, USA) from the skin samples, the 16S rRNA gene amplicon sequencing (16S analysis) method [18] was used to analyze the microbiome community [19] by following the protocol of the research team’s methodology used in the former analysis of oral microbiome of athletes [4].

Sequencing data were analyzed using bioinformatic methods and compared against international databases to evaluate the composition, species richness, and functional properties of the skin microbiome. All steps—including sample collection, laboratory processing, and data analysis—were performed in accordance with strict quality assurance protocols to avoid contamination and ensure reproducibility.

### 2.4. Statistical Analyses

Principal coordinate (PC) analysis was used to visualize the microbiome profile of the studied subgroups—samples with similar features are supposed to be located in a distinct area of the PC ordination plot when the studied characteristic affects the taxonomic diversity. The orthogonal PC axes (Axis.1 and Axis.2) represent the maximum amount of variation in descending order.

The within-group distances were compared to the between-group distances by using an ordination test. The permutational multivariate analysis of variance (PERMANOVA) was used to estimate the similarities between the groups for differential abundance testing, while the relative abundances by sex and athlete/non-athlete division were compared by univariate analysis of relative abundance of genera was performed with ANOVA tests (White’s correction for heteroscedastic data). The khi2 test was used to study the differences/similarities in the relative abundance of taxa between the subgroups. Effect sizes were categorized into small, medium, or large based on Cohen’s criteria (d ≤ 0.10: a small effect; 0.10 < d ≤ 0.25: a medium effect; 0.25 < d ≤ 0.40: a large effect). Hypotheses were tested at the 5% level of random error. The SPSS v. 29 software was used to do the statistical analysis.

### 2.5. Ethical Approval Information

All the participants provided written informed consent prior to inclusion in the study. The study protocol was approved by the institutional ethics committee (TE-KEB/17/2023). The research was supported by grants TEKA2023-141750 and TEKA2024-143061. The examinations were carried out in accordance with the Declaration of Helsinki, revised in 2013.

## 3. Results

In the total sample of athlete and non-athlete youths (aged between 16 and 22 years), it could be stated that *Enhydrobacter*, *Staphylococcus*, *Corynebacterium*, *Acinetobacter*, and *Cutibacterium* genera formed 56% of their skin microbiome community (Table 1). *Enhydrobacter* and *Staphylococcus* were the most abundant genera across samples, comprising 16.2% and 11.9% of all the sequence reads, respectively. Besides these two most abundant genera, *Acinetobacter*, *Cutibacterium*, *Corynebacterium*, *Micrococcus*, *Paracoccus*, *Psychrobacter*, and *Streptococcus* were also important members of the community; their relative abundance ranged between 4.7% and 9.7% in the studied samples.

**Figure 1:**
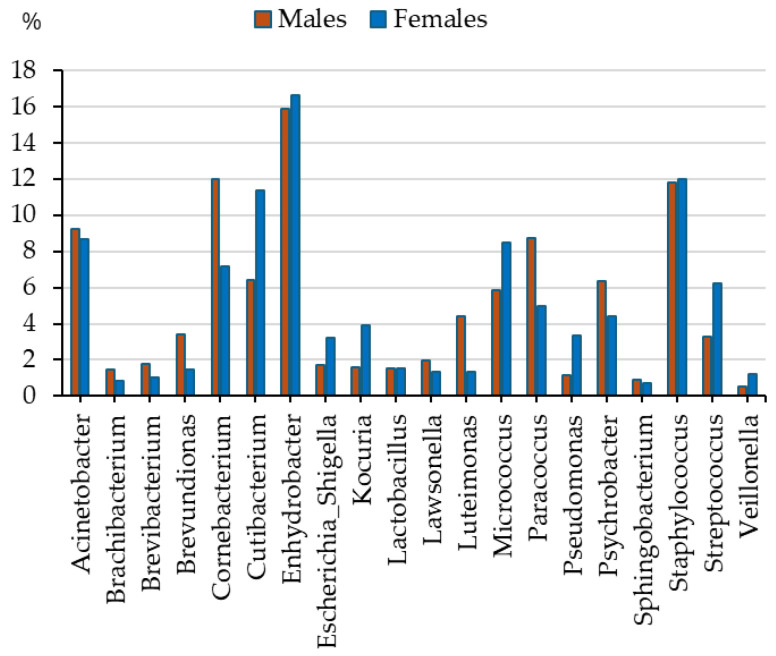
The relative abundances (%, mean) of the most frequent taxa (genus level—taxonomy rank abundances, taxa with a relative abundance > 0.5% are presented) in the skin microbiome of youth by sexes (khi2 test: *p* < 0.05).

By considering the bacterium contents at the genus level by sex, a significant difference (khi^2^ test, *p* < 0.05) of relative abundance of taxa could be detected between males’ and females’ bacterial flora. In the case of the *Corynebacterium*, *Luteimonas*, *Paracoccus*, and *Psychrobacter* genera, males tend to have higher relative abundances, while in the case of the *Cutibacterium*, *Kocuria*, *Micrococcus*, *Pseudomonas*, and *Streptococcus* genera, females had higher relative abundances (Figure 1).

**Figure 2:**
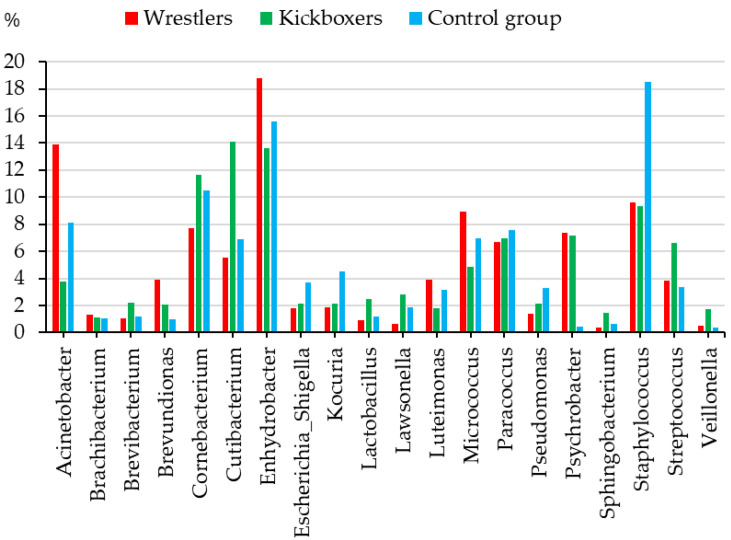
The relative abundances of the most frequent taxa (genus level—taxonomy rank abundances, taxa with a relative abundance > 0.01% are presented) in the skin microbiome of youth by athlete/non-athlete division (wrestlers, kickboxers, and control group; khi2 test: *p* < 0.05).

The higher predominance of the *Cutibacterium* and *Streptococcus* genera and the low frequency of *Enhydrobacter* in kickboxers, while the frequent predominance of the *Acinetobacter*, *Enhydrobacter*, and *Micrococcus* genera in wrestlers compared to the other two subgroups is recognizable in Figure 2 (khi^2^ test, *p* < 0.05). The *Psychrobacter* genus was very rare in the control group compared to the athlete subgroups, while the relative abundance of *Staphylococcus* was higher in athletes than in the control group. To confirm this pattern of differences among the subgroups, principal coordinate analysis of the abundances of the most frequent taxa was carried out on the genus level.

**Figure 3:**
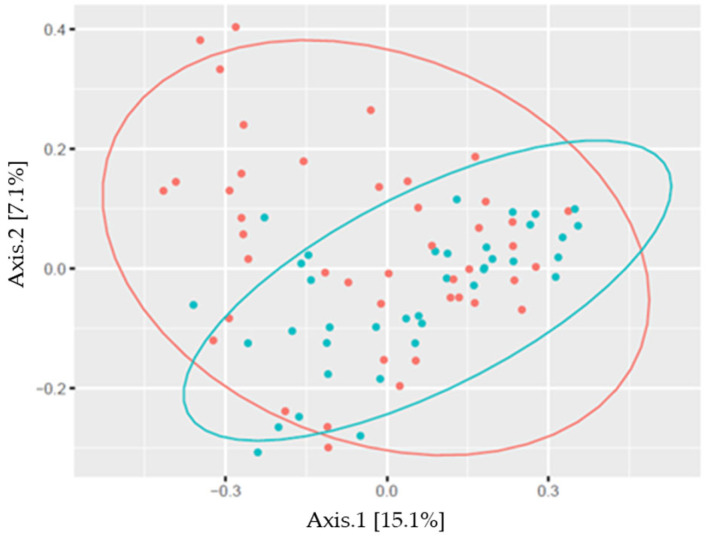
Beta diversity plot (principal coordinate analysis; distance calculation was carried out using Bray–Curtis method) by sexes (●: females, ●: males; %: the variability explained by the axis).

**Figure 4:**
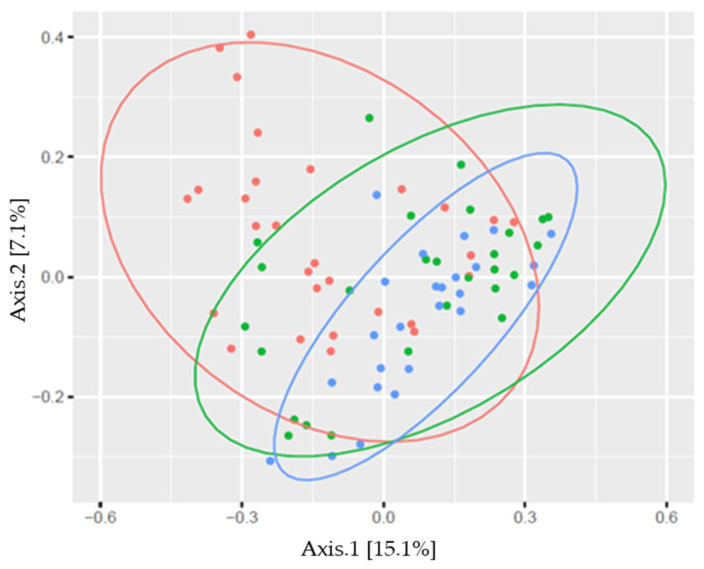
Beta diversity plot (principal coordinate analysis; distance calculation was carried out using Bray–Curtis method) in wrestlers (●), kickboxers (●), and subjects belonging to the control group (●; %: the variability explained by the axis).

The skin microbiome profile significantly differed between males and females, and between athletes and non-athletes in the studied sample (Figure 3 and Figure 4, Table 2).

By comparing males and females, it could be stated that males revealed significantly higher relative abundance (*p* < 0.05, although the analyses revealed in significant differences between the subgroups, the small R^2^ values suggested that small shifts in microbial composition of subgroups accounted only for a small fraction of the total variation) of the following genera (Table 3): *Brevundimonas*, *Corynebacterium*, *Luteimonas* (4.4% vs. 1.4%), *Paracoccus*, and *Psychrobacter* (Table 2), while they had significantly lower relative abundance of the genera *Cutibacterium*, *Escherichia-Shigella*, *Kocuria*, *Micrococcus*, *Pseudomonas*, and *Streptococcus* (Table 2).

Wrestlers had a higher relative abundance of the *Acinetobacter* and *Brevundimonas* genera than in kickboxers or in the members of the control group, while wrestlers and kickboxers had higher relative abundance of *Micrococcus* and *Enhydrobacter* genus than the control group members. The common abundance of the *Escherichia–Shigella*, *Kocuria*, and *Staphylococcus* genera was higher in the control group than in the two subgroups of athletes, while the relative abundance of the *Corynebacterium*, *Cutibacterium*, and *Streptococcus* genera was higher in kickboxers than in wrestlers or members of the control group (Figure 2, Table 2).

The composition of skin microbiome by sports also revealed significant differences: wrestlers had a different composition than kickboxers or youth belonging to the control group, and kickboxers’ microbiome composition did not differ from the control group composition in the PC analysis (Figure 4, Table 2). The analysis showed an apparent pattern of group-specific clustering in both by considering both the sex of the subjects (Figure 3) and the sport type comparison (Figure 4).

## 4. Discussion

### 4.1. Commensals—Healthy Skin Microbiome Representatives in Youth

As expected, the main representatives in the healthy microbiome community of human skin (e.g., *Staphylococcus*, *Corynebacterium*, *Acinetobacter*, and *Cutibacterium*) were the most frequent genera in the studied group of youth. These key members of the skin microbiome play an important part in the defense against pathogens, control of inflammation, the training of the immune system, and microenvironment regulation.

### 4.2. Pathogens Among Skin Microbiome Representatives in Youth

Significant differences were found in the relative abundance of both representatives of normal human microbiome communities and human pathogens between female and male athletes and in the comparison of athletes and non-athletes, too.

#### 4.2.1. Sex Differences in Skin Microbiome Profile of Youth

The present analysis of skin microbiome profile in youth revealed similar results as previous studies in the identification of the dominant members of the bacterial community: more abundant genera in males were *Brevundimonas* (most prevalent causes of cutaneous infections) [20], *Corynebacterium* (representative of normal human microbiome, but it is evidenced that *Corynebacterium* spp. are associated with diseases of the respiratory tract) [21], *Paracoccus* (opportunistic human pathogen, associated with several infections, e.g., conjunctivitis) [22], *Luteimonas* (it is evidenced that *Luteimonas* spp. are associated with skin illnesses, e.g., psoriasis) [23], and *Psychrobacter* (may be a member of the normal human microbiota, but it is evidenced that *Psychrobacter* spp. are related with wound infections) [24], while the *Cutibacterium* (has important role in the maintenance of healthy skin but it is also an opportunistic pathogen of the inflammation in acne vulgaris, the chronic skin disease) [25], *Kocuria* (pathogen of skin and soft tissue infections) [26], *Pseudomonas* (pathogen of infections of the skin, eyes, and ears) [27], *Escherichia–Shigella* (can cause bacterial dysentery) [28], *Micrococcus* (*Micrococcus* spp. are associated with various infections, e.g., bacteremia), and *Streptococcus* (*Streptococcus* spp. can cause several disorders, e.g., skin infections) [29] genera were found in higher relative abundance in females than in males. The factors that can result in these sexual differences in the skin microbiome profile of youth could be differences in hygiene protocols, clothing choices, immune responses to microbial infections, or different hormonal influences [30,31] between males and females; however, to obtain more information about these factors, a future microbial study has been planned, since the present studied did not collect information about these environmental factors.

#### 4.2.2. Athlete–Non-Athlete Differences in Skin Microbiome Profile of Youth

By considering the differences among wrestlers, kickboxers, and members of the non-athlete subgroup, the following could be stated:

(1) The genera Acinetobacter (species are often opportunists in hospitalized patients with impaired immunity, but were also found in contact sports athletes’ skin microbiome indicating the dysbiosis of the wrestlers’ skin) [14] and Brevundimonas (spp. causing infections in immunosuppressed people) [32] had higher relative abundance in wrestlers than members of the other two subgroups—indicating their increased risk for skin infections.
(2) The *Micrococcus* (species are members of the normal skin microbiome; however, they can lead to skin infections in immunocompromised people) [33] and Enhydrobacter (it was evidenced that correlated with the cheek moisture) [34] genera were found in higher abundances in both wrestlers and kickboxers than in non-athletes.
(3) The *Corynebacterium*, *Cutibacterium*, and *Streptococcus* (infection agents of the skin and the respiratory tract) genera were found in higher relative abundance in kickboxers than in wrestlers or non-athletes (opportunistic pathogen, correlates with skin moisture content) [35].
(4) The *Kocuria* (pathogen of skin and soft tissue infections), *Staphylococcus* (*Staphylococcus* spp. may cause skin infections (e.g., abscesses and boils, cellulitis, or folliculitis), and *Escherichia–Shigella* (causing infections in the gastrointestinal tract) genera were less represented in athletes than in non-athletes.
(5) Pathogens in wrestlers’ and kickboxers’ skin microbiome and missing from the microbiome of the control group members’ sample were as follows:
  *Aliterella* sp. (in 29% of athletes)—causing skin infections;
  *Arthrobacter* (in 29% of athletes)—skin infections;
  *Brucella* (in 23% of athletes)—humans can acquire the disease through direct contact with infected animals, consuming animal products, symptoms: fever, fatigue, joint and muscle pain;
  *Empedobacter* sp. (in 39% of athletes) skin infection originating from environmental sources;
  *Peptostreptococcus* (in 14% of athletes)—can become pathogenic under certain conditions, causing skin and other skin tissue infections.

Only very few microbial studies have been conducted in combat sports athletes to explore the microbiome community of their skin. Other studies confirmed that due to direct and indirect (coughing or sneezing) contact transmissions, combat sports athletes are the most vulnerable athletes to skin infections caused by the members of the skin microbiome community. Mennitti et al. [36] identified the *Pseudomonas*, *Acinetobacter*, *Staphylococcus*, *Corynebacterium*, and *Micrococcus* genera as predominant genera on surfaces within the facilities where physical activities occur in combat sports—that result corresponds to the most frequent genera in the skin microbiome of combat sports athletes. The comparison of the results of the present study with results of former microbial studies revealed similarities and differences in the profile of combat sports athletes: the prevalence of the *Bacillus* and *Pseudomonas* genera in the wrestler group was not as high as in the study of Russian combat sports athletes [12], while the microbial community of the skin in combat sports athletes were formed by the same participants (e.g., *Kocuria*, *Staphylococcus*, *Enhydrobacter*, *Micrococcus*, and *Acinetobacter*). The present study identified opportunistic pathogens, community members causing skin infections, and indicators of skin microbiome dysbiosis.

In light of the former and present results of the skin microbial profile of combat athletes, the appropriate skincare product selection is of high importance for combat sports athletes [37] to decrease risk for skin infections, contact-related contaminations.

## 5. Conclusions

Sexual dimorphism could be found in the composition of the skin microbiome community of youth. Genera causing nosocomial and skin infections and respiratory diseases were more frequent in males, while genera causing skin inflammations and infections were found in higher relative abundance in females. The underlying mechanisms of sexual differences in the prevalence of infections and inflammatory diseases in the different tissues in humans [38,39] are still not fully explored. Sexual differences in immune responses and disease susceptibility in skin have already been reported in mice (through an androgen-ILC2-dendritic cell axis mediation) [40]. Besides the immune and neuroendocrine differences, the different skin microbiome may result in the skin health status differences between the two sexes. It is supposed that androgens suppress ILC2 (type 2 innate lymphoid cell) activity, hormonal effect of which (through the level of ILC2-produced cytokines) reduces the number and function of dendritic cells in the skin of males.

The athlete vs. non-athlete comparison also revealed differences, both (1) the pattern of relative abundances of participants in the skin microbiome community found in athletes’ and non-athletes’ skin samples, and (2) the pathogens found only in the athletes’ microbiome indicated an increased risk for skin infections in contact sports athletes. Wrestlers’ microbiome community differed from the non-athletes’ microbiome, while kickboxers revealed a similar microbiome profile to the microbiome profile of members in the non-athlete control group. Kickboxing is a typical striking-only martial art, allowing no grappling or fighting on the ground, while in wrestling, takedowns, grappling, and ground battle are possibilities of fighting. The sportswear of the two studied sport types is also different: in kickboxing, the sportswear covers a bigger portion of the body, and the proportion of free skin surface is smaller in this sport. These two factors could lead to the differences and similarities of the three studied groups of youths’ skin microbiome, but these differences need further investigation. The approximately 30% ratio of wrestlers and kickboxers compared to 0% ratio in the members of the control group with bacteria causing skin infection (*Aliterella*, *Arthrobacter*, and *Empedobacter* spp.) is alarming, demanding regular monitoring and screening of the skin status in these types of martial arts.

Our results confirmed the conclusions of former studies of infectious diseases in contact sports [41]: it is of high importance in contact sports to minimize the risk of infection transmission from direct skin-to-skin contact to maintain the skin and general health status of athletes and to minimize their time lost from competition. Frequent skin-to-skin contact, physical overload, and a weakened immune system altogether can lead to an increased level of susceptibility to infections in athletes in contact sports.

Based on the identified microbiome community members exposing combat sports athletes to potential health risk, it is highly recommended to regularly seek a specialist’s suggestion to prevent skin infections caused by direct and indirect contact microbial transmissions.

## 6. Limitations of the Study

The main limitation of this study was the financial limitation: only a limited number of microbiome samples were collected and examined in the cross-sectional study—the size of the subsamples should be increased in the future. The observed differences indicate only associations, but not causality, given the nature of the design and sample collection.

A longitudinal study could help us to study the main influencing factors of skin microbial composition in combat sports athletes. The presented study faced other challenges, subjected to limitations such as the uncontrolled confounding factors (e.g., diet, personal care products, and personal and environmental hygiene of the athletes and control group—only partial information was collected about these factors, as the personal interviews did not cover all the necessary questions in this aspect).

## Figures and Tables

**Table 1:** Mean relative abundance (SD) of the most frequent bacterial genera in the skin microbiome of the total sample (males vs. females, athletes vs. control group; taxa with a relative abundance ≥ above 1% are presented; *: commensal genera, the other genera are both commensal and pathogenic).

Bacterial Genus	Males	Females	Athletes	Controls	Together
*Enhydrobacter* *	15.9 (2.3)	16.6 (3.9)	16.3 (1.8)	15.6 (1.2)	16.2 (2.3)
*Staphylococcus*	11.8 (3.6)	12.0 (1.7)	9.5 (1.2)	18.5 (3.2)	11.9 (2.0)
*Corynebacterium*	12.0 (1.6)	7.2 (1.0)	9.6 (1.1)	10.5 (1.9)	9.7 (1.0)
*Acinetobacter*	9.3 (2.5)	8.7 (1.8)	9.0 (1.7)	8.1 (1.2)	9.0 (1.5)
*Cutibacterium*	6.4 (1.8)	11.4 (2.8)	9.7 (1.7)	6.9 (1.8)	8.8 (1.7)
*Micrococcus*	5.8 (1.0)	8.5 (0.9)	7.0 (1.0)	7.0 (1.6)	7.1 (0.9)
*Paracoccus*	8.8 (1.5)	5.0 (0.9)	6.8 (0.9)	7.6 (1.2)	7.0 (0.9)
*Psychrobacter*	6.3 (1.3)	4.4 (1.9)	7.3 (1.5)	0.4 (0.2)	5.4 (1.3)
*Streptococcus*	3.3 (1.2)	6.2 (2.8)	5.2 (1.9)	3.4 (1.2)	4.7 (1.6)
*Luteimonas* *	4.4 (0.9)	1.4 (0.7)	2.9 (0.6)	3.2 (1.9)	3.0 (0.6)
*Kocuria*	1.6 (0.5)	3.9 (1.1)	2.0 (0.4)	4.5 (1.6)	2.7 (0.5)
*Brevundimonas*	3.4 (0.9)	1.5 (0.3)	3.0 (0.6)	1.0 (0.8)	2.5 (0.5)
*Escherichia-Shigella*	1.7 (0.5)	3.2 (0.7)	1.9 (0.4)	3.7 (1.1)	2.4 (0.4)
*Pseudomonas*	1.2 (0.3)	3.4 (0.4)	1.8 (0.2)	3.3 (0.8)	2.2 (0.2)
*Lawsonella*	1.9 (0.5)	1.4 (0.4)	1.7 (0.4)	1.8 (0.8)	1.7 (0.3)
*Lactobacillus*	1.5 (0.8)	1.5 (0.4)	1.7 (1.1)	1.2 (0.3)	1.5 (0.4)
*Brevibacterium*	1.8 (0.9)	1.1 (0.5)	1.6 (0.6)	1.2 (0.4)	1.5 (0.6)
*Brachybacterium* *	1.5 (0.2)	0.8 (0.3)	1.2 (0.2)	1.1 (0.3)	1.2 (0.2)

**Table 2:** Significance level (values in bold indicate significant differences) of ANOVA by sex (males: M, females: F; ^m^: males had higher relative abundance, ^f^: females had higher relative abundances) and wrestler–kickboxer–control group comparison (W–KB–C; ^w^: wrestler had the highest relative abundance, ^kb^: kickboxers had the highest relative abundance, and ^c^: control group had the highest relative abundance), respectively, on the relative abundance of bacteria (taxa with a relative abundance ≥1% are presented + *Brachybacterium*: one of the 20 most frequent taxa and revealed significant difference among the subgroups).

	M-F	Effect Size	W-KB-C	Effect Size
*Acinetobacter*	0.384	0.066 (small)	**0.002** ^w^	0.145 (medium)
*Brachybacterium*	**0.047** ^m^	0.378 (medium)	0.559	0.015 (small)
*Brevibacterium*	0.126	0.257 (medium)	0.356	0.026 (small)
*Brevundimonas*	**0.015** ^m^	0.493 (large)	**0.009** ^w^	0.114 (medium)
*Corynebacterium*	**0.009** ^m^	0.538 (large)	0.492	0.018 (small)
*Cutibacterium*	0.063	0.346 (medium)	**0.040** ^c^	0.080 (small)
*Enhydrobacter*	0.487	0.008 (small)	0.218	0.038 (small)
*Escherichia-Shigella*	**0.008** ^f^	0.551 (large)	0.172	0.044 (small)
*Kocuria*	**0.020** ^f^	0.466 (large)	0.318	0.029 (small)
*Lactobacillus*	0.495	0.003 (small)	0.608	0.013 (small)
*Lawsonella*	0.114	0.270 (medium)	**0.005** ^kb^	0.127 (medium)
*Luteimonas*	**0.011** ^m^	0.522 (large)	0.290	0.031 (small)
*Micrococcus*	**0.050** ^f^	0.359 (medium)	**0.008** ^w^	0.115 (medium)
*Paracoccus*	**0.006** ^m^	0.580 (large)	0.900	0.003 (small)
*Pseudomonas*	**0.004** ^f^	0.597(large)	0.461	0.020 (small)
*Psychrobacter*	0.206	0.184 (medium)	**0.028** ^w^	0.088 (small)
*Sphingobacterium*	0.377	0.070 (small)	0.422	0.022 (small)
*Staphylococcus*	0.468	0.018 (small)	0.178	0.043 (small)
*Streptococcus*	**0.032** ^f^	0.313 (large)	0.325	0.028 (small)
*Veillonella*	0.194	0.193 (medium)	0.299	0.031 (small)

**Table 3:** Results of permutational multivariate analysis of variance (PERMANOVA) examining microbiome profile based on the 16S rRNA gene for total bacterial and amplicon sequence variants’ bacterial degraders (W: wrestlers, KB: kickboxers, and C: control group).

	Df	Sum sqs	F	R^2^	*p*
Sex	1	0.594	2.054	0.025	0.005
Sports					
W-KB	1	1.047	4.000	0.069	**<0.001**
W-C	1	1.162	4.427	0.078	**<0.001**
KB-C	1	0.400	1.287	0.025	0.099

Df: degrees of freedom; Sum sqs: sum of squares; F: variance ratio; *p*: level of significance.

## Data Availability

The datasets generated during and/or analyzed during the current study are available from the corresponding author on reasonable request. The data are not publicly available due to privacy and ethical restrictions.
